# Supplementary material for: Deletion of Aurora kinase A prevents the development of polycystic kidney disease in mice
Source: Nat Commun. 2024 Jan 8;15:371. doi: 10.1038/s41467-023-44410-9 (PMC10774271; doi:10.1038/s41467-023-44410-9)
Supplement: Supplementary file 1 — Supplementary Information [file 41467_2023_44410_MOESM1_ESM.pdf]

Supplementary Tables:

Supplementary Table 1: List of Antibodies, Lectins and Stains

Antibodies, Lectins and Stains	Source	ID
Biotinylated Dolichos Biflorus Agglutinin (DBA) 1:250 (IF)	Vector Laboratories	B-1035, RRID:AB_2314288
Biotinylated Lotus Tetragonolobus Lectin (LTL) 1:250 (IF)	Vector Laboratories	B-1325, RRID:AB_2336558
Mouse anti-Acetylated tubulin 1:300 (IF)	Sigma-Aldrich	T6793, RRID:AB_477585
Rabbit anti-AKT 1:100 (IF) 1:1,000 (WB)	CST	#9272, RRID:AB_329827
Rabbit anti-pAKT (T308) 1:100 (IF)	Abcam	ab38449, RRID:AB_722678
Rabbit anti-pAKT (T308) 1:1000 (WB)	CST	#2965, RRID:AB_2255933
Rabbit anti-pAKT (S473) 1:100 (IF) 1:1,000 (WB)	CST	#4060, RRID:AB_2315049
Mouse anti-AURKA (IAK1) 1:100 (IF), 1: 2,000 (WB)	BD Bioscience	610939, RRID:AB_398252
Rabbit anti-pAURKA/B/C (T288) 1:1000 (WB)	CST	#2914, RRID:AB_2061631
Rabbit anti-actin 1: 5,000 (WB)	Sigma-Aldrich	A2066, RRID:AB_476693

Mouse anti-g-H2AX 1:200 (IF) 1:1000 (WB)	Abcam	ab22551, RRID:AB_447150
Rabbit anti-Ki67 1:500 (IF)	Abcam	ab16667, RRID:AB_302459
Rabbit anti-p53 1:250 (IF)	Lecia Biosystems	p53-CM5p, RRID:AB_2744683
Rabbit anti-Pericentrin 1:500 (IF)	Biolegend	923701, RRID:AB_2565440
Rabbit anti-PDK1 (PDK1) 1:100 (IF)	Thermofisher Invitrogen	PA5-79801, RRIF:AB_2746916
Rabbit anti-THP (UMOD) 1:50-100 (IF)	SCBT	H-135 (discontinued), RRID:AB_2212378
Rabbit anti-UMOD (THP) 1:500 (IF)	Abcam	ab207170
Mouse anti-Uroplakin-III 1:50 (IF)	Progen	651108, RRID:AB_1543140
Mouse anti-V5 clone V5-10 1:1000 (WB)	Sigma-Aldrich	V8012, RRID:AB_261888
Rabbit anti-V5 1:100 (IF)	CST	#13202, RRID:AB_2687461
Goat anti-V5 conjugated agarose bead (IP)	Abcam	Ab1229, RRID:AB_308681

Alexa Fluor® donkey anti-mouse IgG (H+L) 488 1:600 (IF)	Molecular Probes	A21202, RRID:AB_141607
Alexa Fluor® donkey anti-mouse IgG (H+L) 555 1:600 (IF)	Molecular Probes	A31570, RRID:AB_2536180
Alexa Fluor® donkey anti-mouse IgG (H+L) 647 1:600 (IF)	Molecular Probes	A31571, RRID:AB_162542
Alexa Fluor® goat anti-mouse IgG1 (H+L) 488 1:600 (IF)	Molecular Probes	A21121, RRID:AB_2535764
Alexa Fluor® goat anti-mouse IgG2b (H+L) 555 1:600 (IF)	Molecular Probes	A21147, RRID:AB_2535783
Alexa Fluor® donkey anti-rabbit IgG (H+L) 488 1:600 (IF)	Molecular Probes	A21206, RRID:AB_2535792
Alexa Fluor® donkey anti-rabbit IgG (H+L) 555 1:600 (IF)	Molecular Probes	A31572, RRID:AB_162543
Alexa Fluor® donkey anti-rabbit IgG (H+L) 647 1:600 (IF)	Molecular Probes	A31573, RRID:AB_2536183
Dolichos Biflorus Agglutinin (DBA)- FITC 1:50-100 (IF)	Vector Laboratories	FL-1031, RRID:AB_2336394
Donkey anti-rabbit IgG (H+L) HRP 1:10,000 (WB)	Millipore	AP182P, RRID:AB_92591
Goat anti-mouse IgG (H+L) HRP 1:10,000 (WB)	Millipore	P0447, RRID:AB_2617137
Streptavidin, Alexa Fluor® 488 1:600 (IF)	Molecular Probes	S11223
Streptavidin, Alexa Fluor® 555 1:600 (IF)	Molecular Probes	S21381

Streptavidin, Alexa Fluor® 647 1:600 (IF)	Molecular Probes	S21374
DAPI (4’,6-diamidino-2-phenylindole) 1:5,000 (IF)	DAKO	D1306, RRID:AB_2629482

Supplementary Figures

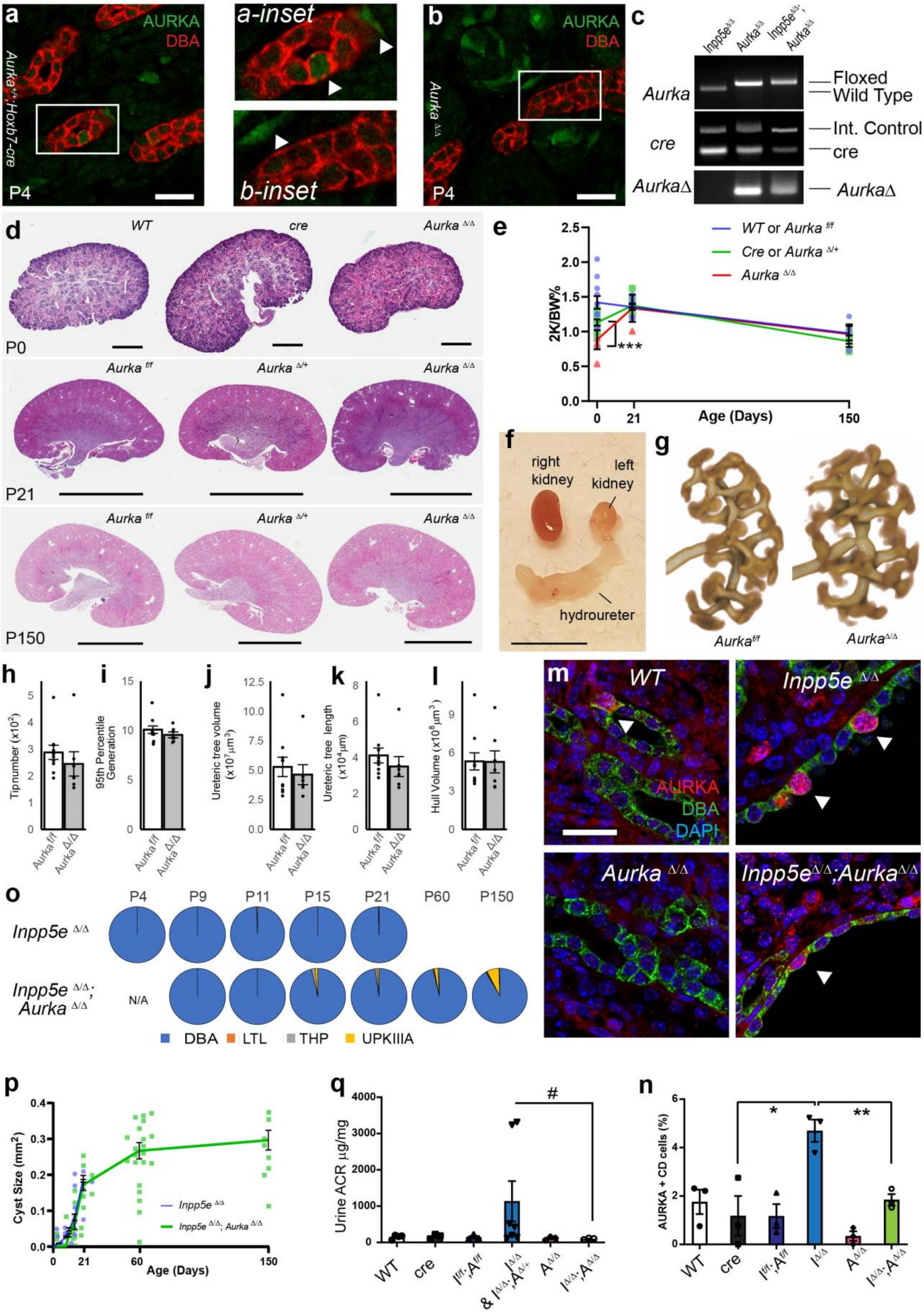

Fig S1: Deletion of Aurka has no kidney phenotype & extended characterisation of neonatal JS PKD mice

(a,b) Kidney sections stained for DBA, AURKA and DAPI in animals of ages and genotypes indicated (scalebar = 20µm). Inset arrowheads indicate AURKA+ve cells. (c) Genomic PCR analysis of *Aurka* wildtype, floxed and $^{\Delta/\Delta}$ loci with *cre* and internal controls. (d) Kidney H&E sections form wild type, *cre, Aurka*$^{\Delta/+}$ and/or *Aurka*$^{\Delta/\Delta}$ mice at indicated ages (scalebar = 0.5 mm at P0, 4 mm P21 and P150). (e) Combined kidney weight over total body weight percentage (2K/BW%) (p = 0.0005). (f) Image of hydroureter and hydronephrosis occasionally observed in *Aurka*$^{\Delta/\Delta}$ mice. (G-L) OPT analysis of E14.5 embryonic kidneys assessing parameters including 3D renders of kidneys (g), tips number (h), branch generations (i), tree volume (j), tree length (k) and kidney hull volume (l). (m) Kidney sections stained for DBA, DAPI and AURKA at P21, Arrowheads indicate AURKA+ve CD cells in cysts (scalebar = 50 µm). (n) Quantification of % AURKA +ve collecting duct cells at P21. (left to right p-values 0.0153, 0.0059). (o) Identity of cyst cell identity following staining with DAPI, DBA and UP3AIII. (p) Quantification of the cyst cross sectional area over time. (r) Quantification of the urine Albumin Creatinine Ratios (ACR) in µg/mg. (p-value = 0.0551). All graph data indicate mean $\pm$ S.E.M. (a-c) n=3, (d-e) n=3-12, (g-l) n=7-10, (m,n) n=3, (o-p) n= 3-23, (q) n= 3-7. Exact n values and data point values provided in Source Data File.

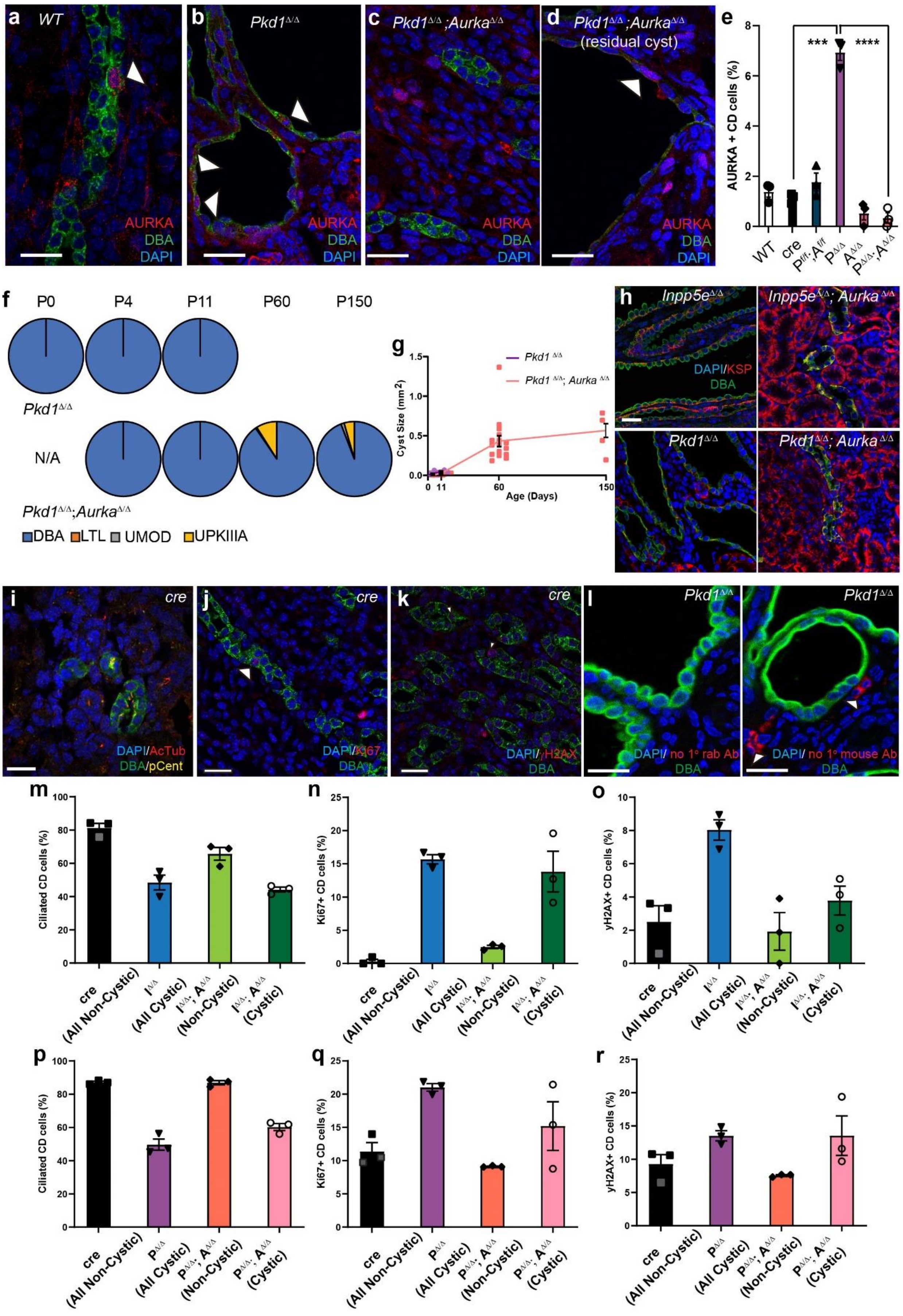

a WT
AURKA
DBA
DAPI
b Pkd1Δ/Δ
c Pkd1Δ/Δ;AurkaΔ/Δ
d Pkd1Δ/Δ;AurkaΔ/Δ (residual cyst)
e
AURKA + CD cells (%)
***
****
WT
cre
Pf/f;Af/f
PΔ/Δ
AΔ/Δ
PΔ/Δ;AΔ/Δ
f P0 P4 P11 P60 P150
Pkd1Δ/Δ
N/A
Pkd1Δ/Δ;AurkaΔ/Δ
DBA LTL UMOD UPKIIIA
g
Pkd1 Δ/Δ
Pkd1 Δ/Δ; Aurka Δ/Δ
Cyst Size (mm2)
Age (Days)
h Inpp5eΔ/Δ
Inpp5eΔ/Δ; Aurka Δ/Δ
DAPI/KSP
DBA
Pkd1Δ/Δ
Pkd1Δ/Δ; Aurka Δ/Δ
i cre
DAPI/AcTub
DBA/pCent
j cre
DAPI/Ki67
DBA
k cre
DAPI/γH2AX
DBA
l Pkd1Δ/Δ
DAPI/ no 1° rab Ab
DBA
Pkd1Δ/Δ
DAPI/ no 1° mouse Ab
DBA
m
Ciliated CD cells (%)
cre (All Non-Cystic)
IΔ/Δ (All Cystic)
IΔ/Δ; AΔ/Δ (Non-Cystic)
IΔ/Δ; AΔ/Δ (Cystic)
n
Ki67+ CD cells (%)
o
γH2AX+ CD cells (%)
p
Ciliated CD cells (%)
cre (All Non-Cystic)
PΔ/Δ (All Cystic)
PΔ/Δ; AΔ/Δ (Non-Cystic)
PΔ/Δ; AΔ/Δ (Cystic)
q
Ki67+ CD cells (%)
r
γH2AX+ CD cells (%)

Fig S2: Extended characterisation of neonatal ADPKD mice

a-d) P4 Immunofluorescent imaging of AURKA expression in WT collecting ducts, *Pkd1*$^{\Delta/\Delta}$ cysts and residual cysts in *Pkd1* $^{\Delta/\Delta}$*; Aurka* $^{\Delta/\Delta}$ mutants but not normal collecting ducts. Arrowheads indicate AURKA+ve cells. e) Quantitation of AURKA expressing collecting duct cells across indicated genotypes at P4. P values from left to right; p=0.0006 and 0.0001. f) Kidney sections stained with DAPI, DBA, THP and LTL to determine cyst identity. g) Quantification of the cyst cross sectional area over time. h) Immunostaining of P21 *Inpp5e*$^{\Delta/\Delta}$, P11 *Pkd1*$^{\Delta/\Delta}$ and rescued counterpart kidney sections for DAPI, DBA and KSP. i-k) P4 immunofluorescent imaging of control *cre* collecting ducts, labelled as indicated. l) P11 secondary only control imaging of *Pkd1*$^{\Delta/\Delta}$ cysts without rabbit or without mouse primary antibody, as indicated. Non-specific staining was low for anti-rabbit IgG secondary antibodies. Anti-mouse IgG secondary antibody did however label occasional interstitial mouse cells (arrowheads) but rarely tubular cells. m-o) P21 *Inpp5e* model data for % Ciliated CD cells, % Ki67 CD cells and % γH2AX CD cells highlighting behaviour of cystic and non-cystic regions in *Inpp5e* $^{\Delta/\Delta}$*; Aurka* $^{\Delta/\Delta}$ mutants. p-r) P11 *Pkd1* model data for % Ciliated CD cells, % Ki67 CD cells and % γH2AX CD cells highlighting behaviour of cystic and non-cystic regions in *Pkd1* $^{\Delta/\Delta}$*; Aurka* $^{\Delta/\Delta}$ mutants. Bars = 25 μm. All graph data indicate mean $\pm$ S.E.M. (a-e) n =3, (f-g) n=3-16, (h-r) n=3. Exact n values and data point values provided in Source Data File.

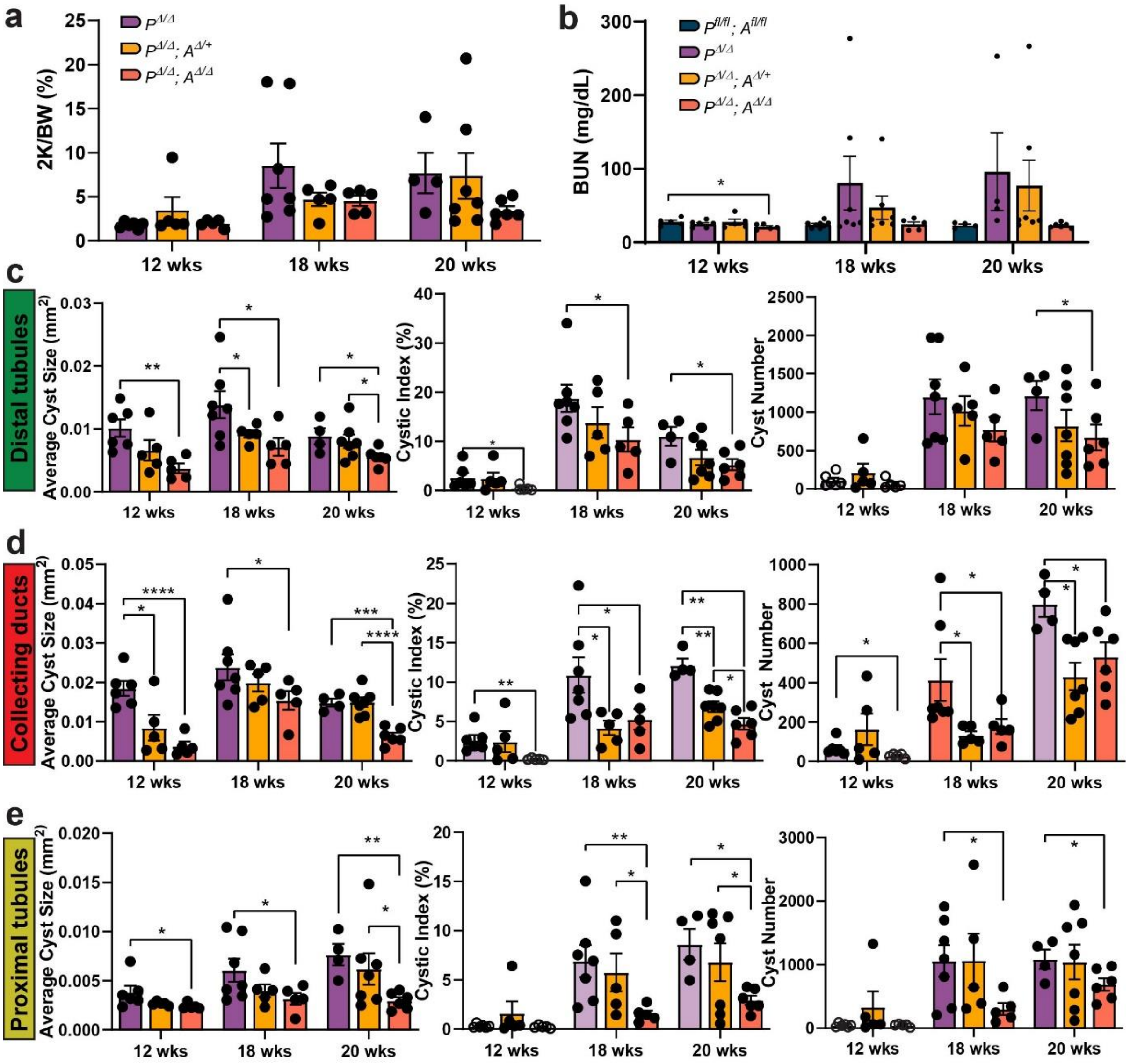

Fig S3: Extended characterisation of adult onset ADPKD mice

(a) 2KW/BW ratios. (b) Assessment of Blood Urea Nitrogen (BUN) as a measure of kidney function mice of indicated genotypes at fixed experimental time point. p=0.0241 (c) The average cyst size, cystic index and cyst number per cross section for tubule cyst identity in distal tubules at 12, 18 and 20-weeks. P values from left to right: average cyst size – 0.0019, 0.0384, 0.0134, 0.0376, 0.0355; cystic index – 0.0384, 0.0238, 0.0248; cyst number 0.0354. (d) The average cyst size, cystic index and cyst number per cross section for tubule cyst identity in collecting ducts at 12, 18 and 20-weeks. P values from left to right: average cyst size – 0.0001, 0.0170, 0.0330, 0.0002, 0.0001; cystic index – 0.0057, 0.0125, 0.0290, 0.0023, 0.0002, 0.0261; cyst number – 0.0283, 0.0194, 0.0365, 0.0021, 0.0129. (e) The average cyst size, cystic index and cyst number per cross section for tubule cyst identity in proximal tubules at 12, 18 and 20-weeks. P values from left to right: average cyst size – 0.0425, 0.0283, 0.0091, 0.0451; cystic index – 0.0079, 0.0462, 0.0159, 0.0456; cyst number – 0.0113, 0.0385. All graphs indicate mean + S.E.M. (a-e) n=4-8. Exact n values and data point values provided in Source Data File.

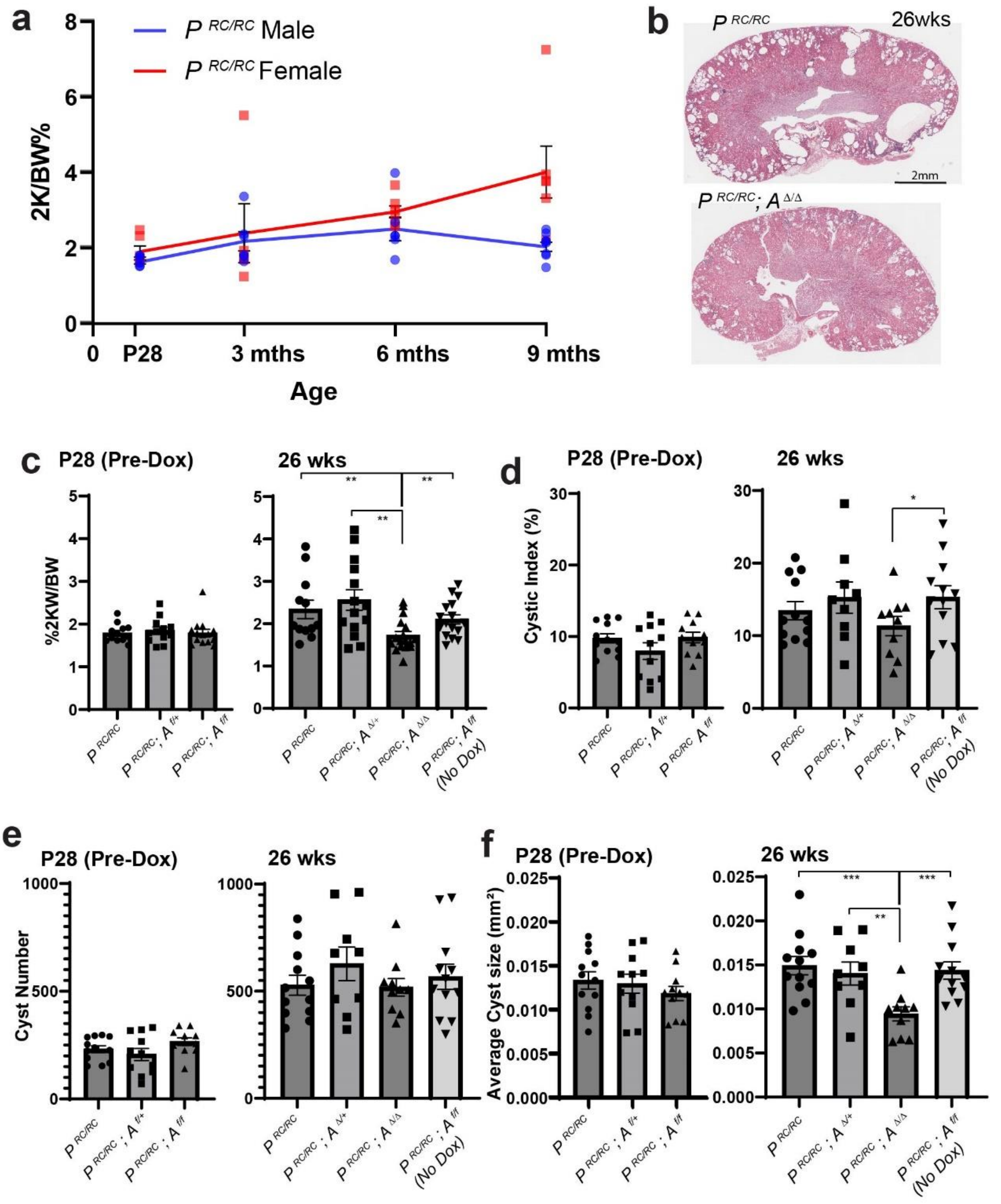

Fig S4: Characterisation of adult onset ADPKD RC mice

(a) 2K/BW ratios of male and female *Pkd1* $^{RC/RC}$ mice over time, showing sex divergence from 6 to 9 mths of age. (b) H&E sections of 26 week old *Pkd1* $^{RC/RC}$ *(P* $^{RC/RC}$*)* with and without *Aurka (A* $^{\Delta/\Delta}$*)* mice. c-f) Quantification of 2K/BW, cystic index, cyst number and size at postnatal day 28 (P28) and 26 weeks of age (22 weeks after doxycycline treatment to delete *Aurka*). (c-d) P values from left to right = 0.0099, 0.0026, 0.0063, 0.0352. (e-f) P values from left to right less than 0.0002, 0.0050, 0.0005. All graphs indicate mean + S.E.M. (a) n=5-8, (b-f) n = 9-14. Exact n values and data point values provided in Source Data File.

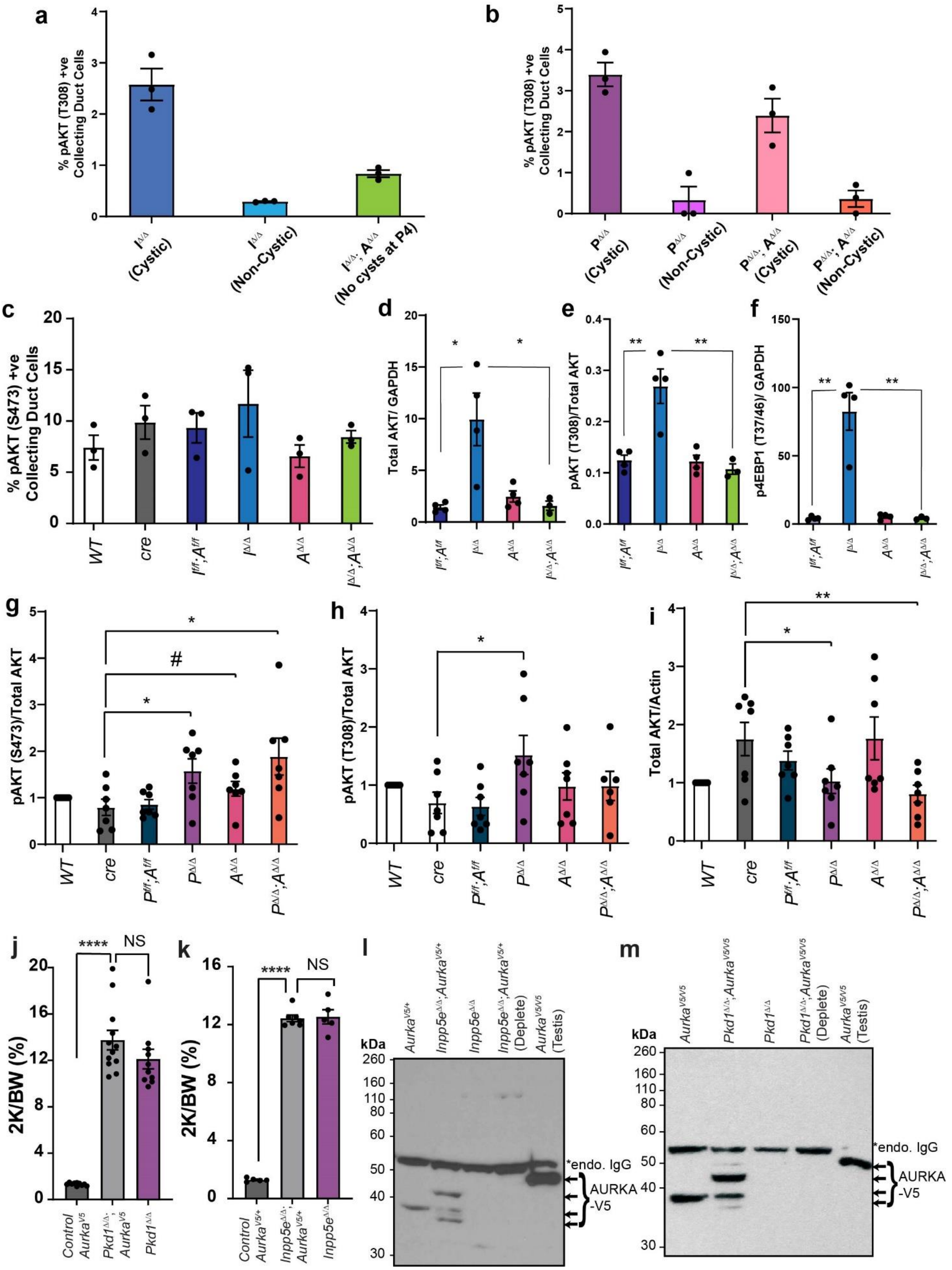

a
% pAKT (T308) +ve Collecting Duct Cells
IΔ/Δ (Cystic)
IΔ/Δ (Non-Cystic)
IΔ/Δ; AΔ/Δ (No cysts at P4)
b
% pAKT (T308) +ve Collecting Duct Cells
PΔ/Δ (Cystic)
PΔ/Δ (Non-Cystic)
PΔ/Δ; AΔ/Δ (Cystic)
PΔ/Δ; AΔ/Δ (Non-Cystic)
c
% pAKT (S473) +ve Collecting Duct Cells
WT
cre
If/f;Af/f
IΔ/Δ
AΔ/Δ
IΔ/Δ;AΔ/Δ
d
Total AKT/ GAPDH
e
pAKT (T308)/Total AKT
f
p4EBP1 (T37/46)/ GAPDH
g
pAKT (S473)/Total AKT
#
h
pAKT (T308)/Total AKT
i
Total AKT/Actin
WT
cre
Pf/f;Af/f
PΔ/Δ
AΔ/Δ
PΔ/Δ;AΔ/Δ
j
2K/BW (%)
****
NS
Control AurkaV5
Pkd1Δ/Δ; AurkaV5
Pkd1Δ/Δ
k
2K/BW (%)
Control AurkaV5/+
Inpp5eΔ/Δ; AurkaV5/+
Inpp5eΔ/Δ
l
kDa
AurkaV5/+
Inpp5eΔ/Δ;AurkaV5/+
Inpp5eΔ/Δ
Inpp5eΔ/Δ;AurkaV5/+ (Deplete)
AurkaV5/V5 (Testis)
*endo. IgG
AURKA -V5
m
kDa
AurkaV5/V5
Pkd1Δ/Δ;AurkaV5/V5
Pkd1Δ/Δ
Pkd1Δ/Δ;AurkaV5/V5 (Deplete)
AurkaV5/V5 (Testis)
*endo. IgG
AURKA -V5

Fig S5: AKT activity and extended COIP data

(a,b) Quantification of % pAKT (T308) +ve collecting duct cells at P4, split by genotype and cystic and non-cystic tubules. (c) Quantification of pAKT (S473) +ve collecting duct cells in *Inpp5e* model at P4. (d) AlphaLISA analysis of Total AKT over total GAPDH from P21 whole kidney lysates from *Inpp5e* model (p=0.0219 for *Inpp5e*$^{\Delta/\Delta}$ kidneys relative to floxed control and p=0.0222 relative to *Inpp5e*$^{\Delta/\Delta}$*;Aurka*$^{\Delta/\Delta}$. (e) AlphaLISA analysis of pAKT (T308) ratio from whole kidney lysates (p=0.0094 for *Inpp5e*$^{\Delta/\Delta}$ kidneys relative to floxed control and p=0.0066 relative to *Inpp5e*$^{\Delta/\Delta}$*;Aurka*$^{\Delta/\Delta}$ kidneys) at P21. (f) AlphaLISA analysis of p4EBP1 (T37/46) over total GAPDH from whole kidney lysates (p=0.0054 for *Inpp5e*$^{\Delta/\Delta}$ kidneys relative to both floxed controls and *Inpp5e*$^{\Delta/\Delta}$*;Aurka*$^{\Delta/\Delta}$ kidneys) at P21. g-i) Densitometry of Western blots of P4 kidney lysates from *Pkd1*$^{\Delta/\Delta}$*;Hoxb7* mice. Western blots represent biological replicates/individual animals with each independent experiment. Each WT control sample within a replicate set was defined as 1. Blots were repaired by probing, stripping and reprobing of the same membrane to generate datasets. g) pAKT (S473)/Total AKT ratio (left to right p=0.0159, p=0.0598, p=0.0175). (h) pAKT (T308)/Total AKT ratio (*Pkd1*$^{\Delta/\Delta}$ vs *cre* p = 0.0295). Single outlier (>Q3+ 1.5 x IQR) removed from *Pkd1*$^{\Delta/\Delta}$*;Aurka*$^{\Delta/\Delta}$ dataset. (i) Total AKT expression level (left to right p=0.0334, p=0.0085). (j,k) Comparison of combined kidney to body weight percentage (2K/BW%) in mice of indicated genotypes (*Aurka* $^{V5/V5}$relative to *Pkd1*$^{\Delta/\Delta}$;*Aurka* $^{V5/V5}$ mice, p value= 6.6 x 10^{-9}. *Aurka* $^{V5/+}$ relative to *Inpp5e*$^{\Delta/\Delta}$;*Aurka* $^{V5/+}$ mice, p value= 5.0 x 10^{-10}). (l,m) Input lysates from P11 (*Pkd1*) and P15 (*Inpp5e*) mouse kidneys of indicated genotypes. Mouse testis from adult *Aurka*$^{V5/V5}$ mice was included as a positive control. Asterisked upper band is mouse endogenous IgG in tissue extracts detected by the anti-mouse IgG secondary antibody used to identify mouse monoclonal anti-V5. All graph data indicate mean $\pm$ S.E.M. (a-c) n=3, (d-f) n=3-4, g-i) n =6-7, (j) n=10-12, (k) n=5-7, (l,m) n=3, NS= not significant. Individual western blot scans, n and data point values provided in supplementary material- Source Data File. All graph data indicate mean $\pm$ S.E.M.

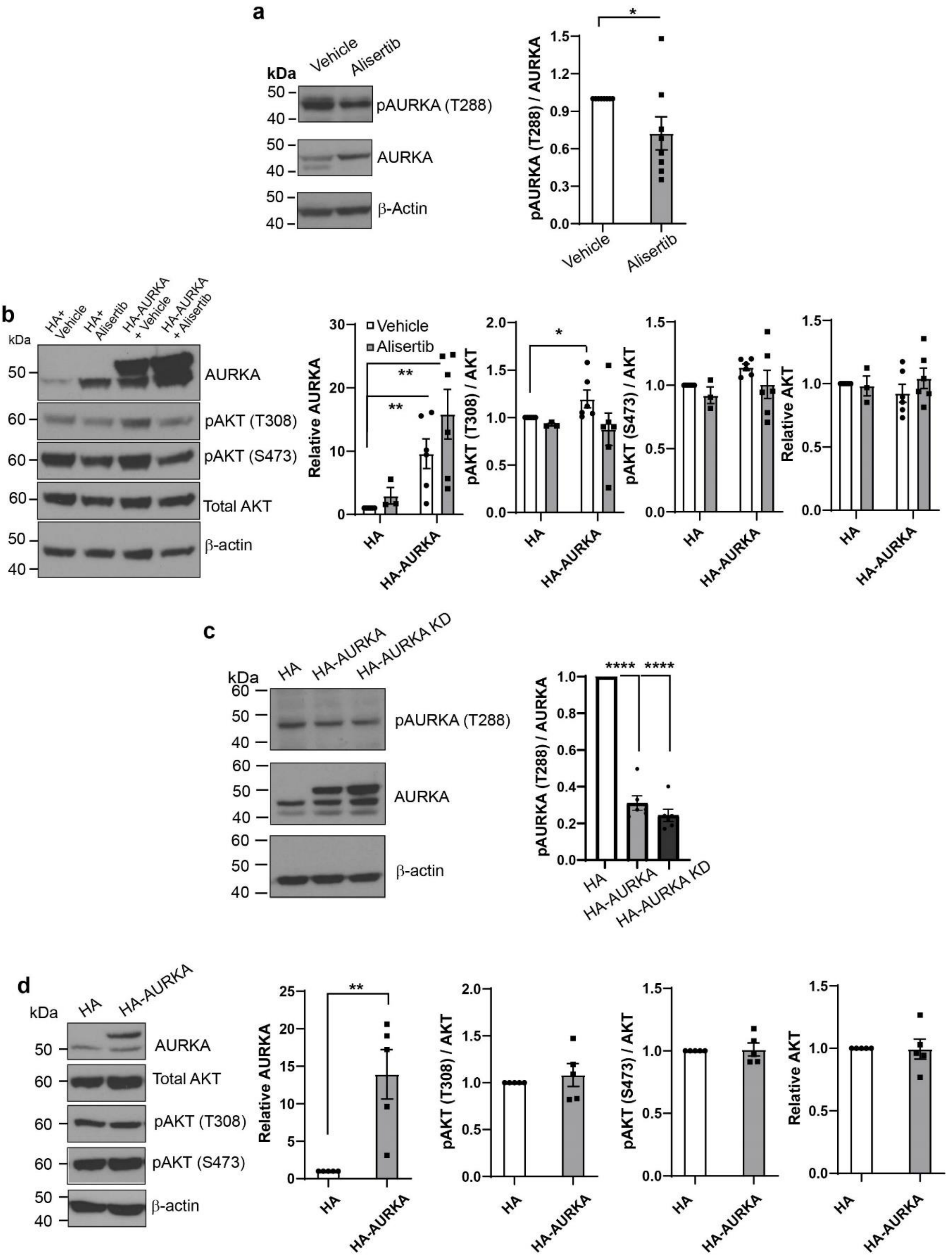

a
Vehicle
Alisertib
kDa
50
40
pAURKA (T288)
AURKA
β-Actin
pAURKA (T288) / AURKA
1.5
1.2
0.9
0.6
0.3
0.0
*
b
HA+ Vehicle
HA+ Alisertib
HA-AURKA + Vehicle
HA-AURKA + Alisertib
kDa
60
AURKA
pAKT (T308)
pAKT (S473)
Total AKT
β-actin
Vehicle
Alisertib
Relative AURKA
30
20
10
0
**
pAKT (T308) / AKT
2.0
1.5
1.0
0.5
pAKT (S473) / AKT
Relative AKT
HA
HA-AURKA
c
HA-AURKA KD
0.8
0.4
0.2
****
d
25
15
5

Fig S6: AURKA regulates pAKT (T308) extended

(a) Western blots and densitometry of mIMCD3 cell lysates treated with Alisertib (1μM) or vehicle for 48 hrs under growth conditions. Alisertib reduces the pAURKA (T288) ratio ($p = 0.0372$, n=8). (b) Western blots and densitometry of mIMCD3 cell lysates transfected with HA or HA-AURKA expression plasmids, with 24 hrs exposure to Alisertib (1μM) or DMSO vehicle. AURKA is over-expressed ($p = 0.0073$, 0.0064). HA-AURKA expression increases pAKT (T308)/total AKT ratio ($p = 0.0455$), this does not occur with 24 hrs of Alisertib exposure. pAKT (S473)/total AKT ratio and relative AKT expression remain similar with Alisertib (n=3-6). (c) Western blots and densitometry of mIMCD3 cell lysates treated with HA, HA-AURKA or HA-AURKA KD expression plasmids, then cultured under growth conditions. Neither HA-AURKA or HA-AURKA KD show significant phosphorylation at T288, whereas only a band consistent with endogenous AURKA does, suggesting over-expressed AURKA is predominantly maintained in a kinase inactive state. HA-AURKA and HA-AURKA KD both reduce relative phosphorylation at T288 over total AURKA levels, p-value = 5.5 x10^-6 and 1.61x10^-6, n=6. (d) Western blots and densitometry of serum starved (24hr) mIMCD3 cell lysates transfected with HA or HA-AURKA expression plasmids probed for AURKA, total AKT, pAKT (T308), pAKT (S473) and actin. AURKA is overexpressed (p-value = 0.0086) but no changes in pAKT (T308)/total AKT, pAKT (S473)/total AKT ratios or relative AKT expression are observed, n=5. Western blots represent biological replicates with each independent experiment set run on a separate gel or two replicates on a gel. Each control sample within a replicate set was then defined as 1. Western membranes were cut to 40-60kDa interval before antibody probing, stripping and reprobing. Due to limits of stripping and reprobing, some replicates for S6b and S6d were probed across repeat gel sets performed in parallel. S6a and S6c were reprobes of the same membrane. Individual western blot scans, n and data point values provided in supplementary material- Source Data File. All graph data indicate mean $\pm$ S.E.M.

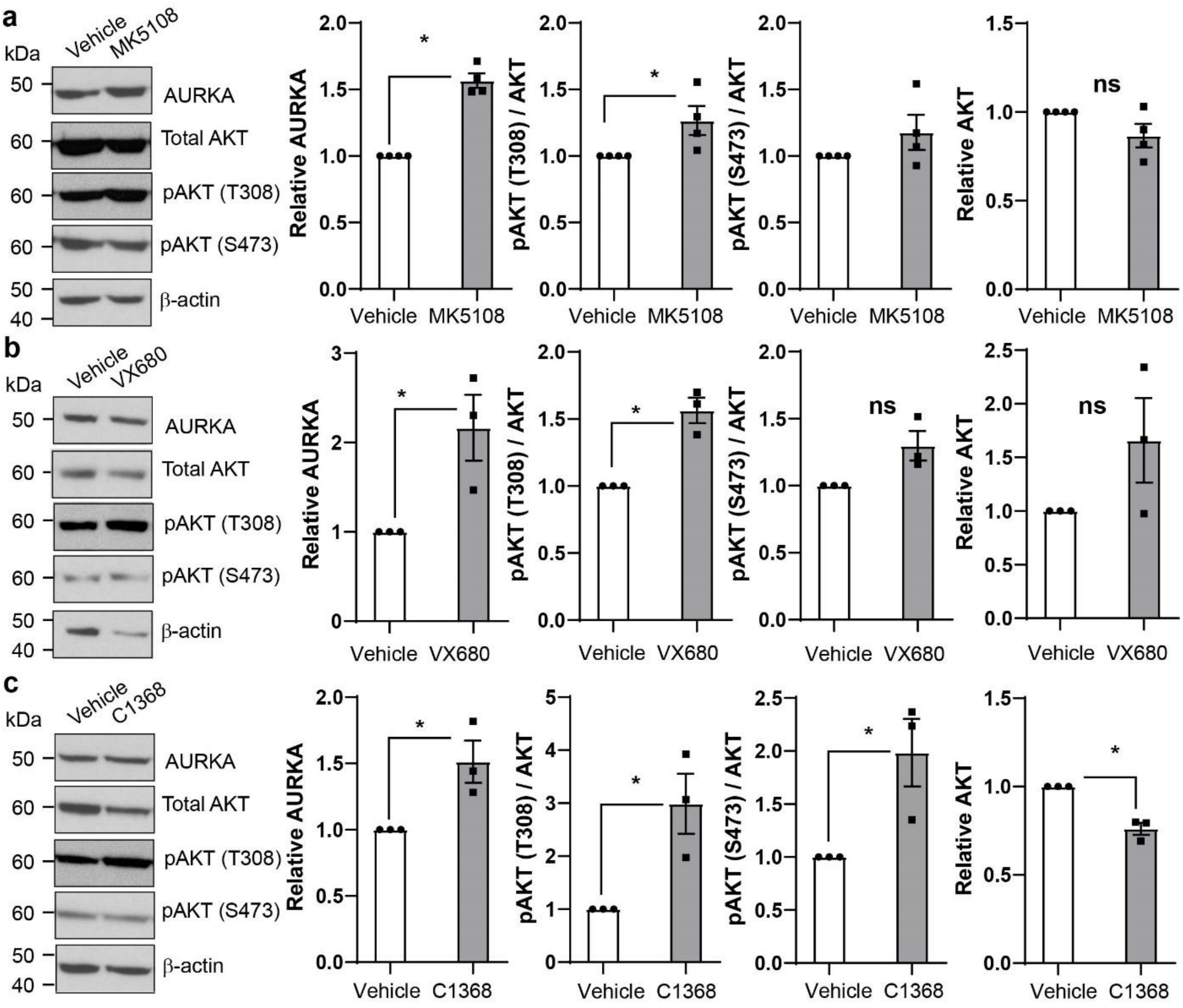

Fig S7: AURKA regulates pAKT (T308) extended Part2

(a-c) Western blots and densitometry of mIMCD3 cell lysates treated with AURKA kinase inhibitors MK5108 (1μM), VX680 (500nM), C1368 (2μm) or vehicle for 48 hrs under growth conditions. All inhibitors promoted AURKA upregulation (p = 0.0009, 0.0464, 0.0420 respectively) and increases in the pAKT (T308)/AKT ratio (p = 0.0464, 0.0135, 0.0361 respectively). In contrast, the pAKT (S473)/AKT ratio was not statistically different with MK5108 and VX680, but altered with C1368 (p=0.0455). Total AKT levels were not statistically different with MK5108 and VX680, but were significantly reduced by C1358 (p<0.0103), MK5108 n=4, VX680 & C1368 n=3. All graph data indicate mean ± S.E.M. Western blots represent biological replicates with each independent experiment set stored and run on the same gels. Each control sample within a replicate was defined as 1. Blots were repaired by probing, stripping and reprobing of the same membrane to generate datasets. Exact n and data point values provided in Source Data File.

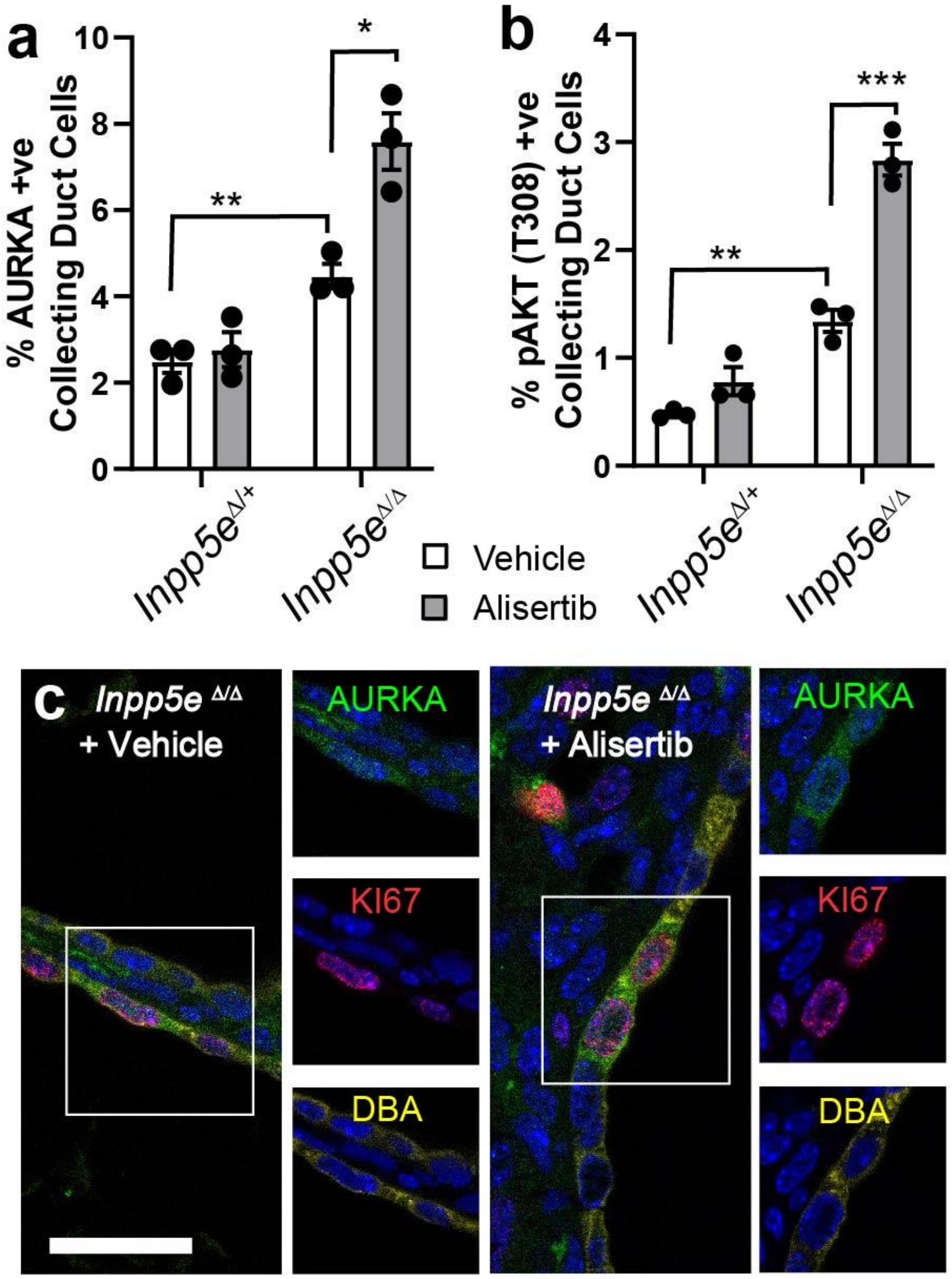

Fig S8: Extended characterisation of Alisertib treatments

(a,b) Quantification of AURKA (a) and pAKT (T308) (b) +ve collecting duct cells. P values from left to right; p=0.0035, p=0.0134, p=0.0051, p=0.0009. (c) Immunostaining of Ki67/AURKA in collecting duct cells marked with DBA during vehicle or Alisertib treatment of P15 *Inpp5e*$^{\Delta/\Delta}$ mice (bar = 25 μm). All graph data indicate mean $\pm$ S.E.M. Abbreviation pT308 = pAKT (T308). White bars = vehicle; Grey bars = Alisertib treatment. a-c) n=3. All data from P15. Exact n and data point values provided in Source Data File.

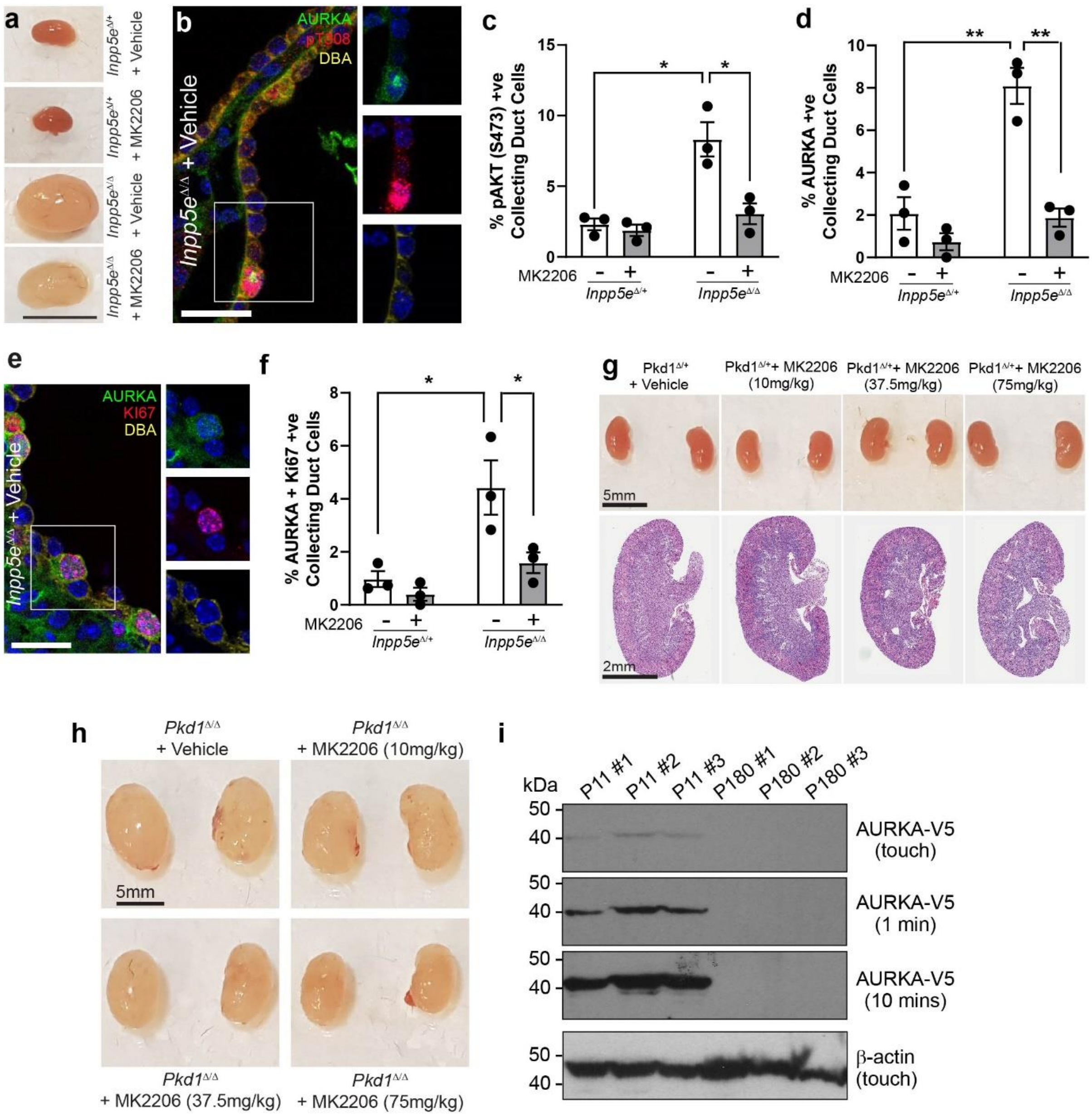

Fig S9: Extended characterisation of MK2206 treatments

(a,g,h) Representative images of mouse whole kidneys or sections from P15 *Inpp5e* or P11 *Pkd1* mice orally treated with vehicle or MK2206. Whole kidney photo bar = 0.5 cm, H&E bar = 2 mm. (b) Staining for AURKA, DBA and pAKT (T308) in mice exposed to vehicle (bar = 25 µm). (c,d) Quantification of the proportion of pAKT (S473) +ve or AURKA+ve collecting duct cells in *Inpp5e*$^{\Delta/\Delta}$ and control mice at P15, following treatment with Vehicle or MK2206 (n=3) p values left to right, 0.0140, 0.0146, 0.0032, 0.0038. (e) Staining for AURKA, DBA and Ki67 in mice exposed to vehicle (bar = 25 µm). (f) Quantification of the proportion of Ki67 or AURKA+ve collecting duct cells in *Inpp5e*$^{\Delta/\Delta}$ and control mice at P15, following treatment with Vehicle or MK2206 (n=3) p values left to right 0.0340, 0.0476. (i) Detection of AURKA in tissue extracts from P11 and 6 month old mouse kidneys of *Aurka*$^{V5/V5}$ mice noting exposure time. (g-h) n =4-12. All graph data indicate mean ± S.E.M. Abbreviation pT308 = pAKT (T308). Exact n and data point values provided in Source Data File.
