# Supplementary material for: Deletion of Aurora kinase A prevents the development of polycystic kidney disease in mice
Source: Nat Commun. 2024 Jan 8;15:371. doi: 10.1038/s41467-023-44410-9 (PMC10774271; doi:10.1038/s41467-023-44410-9)
Supplement: Supplementary file 3 — Description of Additional Supplementary Files [file 41467_2023_44410_MOESM3_ESM.pdf]

Description of Additional Supplementary Files

Supplementary Data 1- RNAseq Differentially Expressed Gene Lists from P4 murine whole kidneys.
Supplementary Data 2- KEGG pathway analysis from RNAseq Gene lists
